# Paradoxical palmoplantar pustulosis induced by secukinumab in a patient with hidradenitis suppurativa

**DOI:** 10.1016/j.jdcr.2026.02.011

**Published:** 2026-02-13

**Authors:** Riam Saleh Alkhamis, Abdulaziz Ahmed Alomari, Alwaleed Mohammed Altuwaijri, Mohammed Abdulaziz Alajlan, Ibrahim Mohammed AlFuraih

**Affiliations:** a Dermatology Department, King Saud Medical City, Riyadh, Saudi Arabia; b College of Medicine, Al-Maarefa University, Riyadh, Saudi Arabia; c College of Medicine, King Saud bin Abdulaziz University for Health Sciences, Riyadh, Saudi Arabia; d Dermatology Department, King Fahad Medical City, Riyadh, Saudi Arabia

**Keywords:** biologic therapy, hidradenitis suppurativa, palmoplantar pustulosis, paradoxical reaction, secukinumab, skin of color

We present a case of palmoplantar pustulosis (PPP) occurring as a paradoxical reaction to secukinumab in a 26-year-old man with long-standing Hurley stage III hidradenitis suppurativa (HS). Following the fifth dose of secukinumab, the patient developed painful, erythematous, scaly plaques with sterile pustules localized to the palms and soles. The clinical presentation was consistent with PPP, and the lesions improved after discontinuation of secukinumab and initiation of adalimumab.

Recognition of paradoxical palmoplantar pustular eruptions during IL-17 inhibitor therapy is essential although rare, as these reactions can clinically resemble other dermatoses. Prompt clinical evaluation is necessary to avoid misdiagnosis and ensure appropriate management.

## Case description

A 26-year-old man with long-standing HS, Hurley stage III, was referred for worsening disease activity. Since 2015, he had experienced recurrent painful nodules, draining sinus tracts, and abscesses involving the axillae, groin, and buttocks. Previous treatments included topical clindamycin, benzoyl peroxide, chlorhexidine washes, and a course of oral isotretinoin, with only minimal to no response.

**Fig 1:**
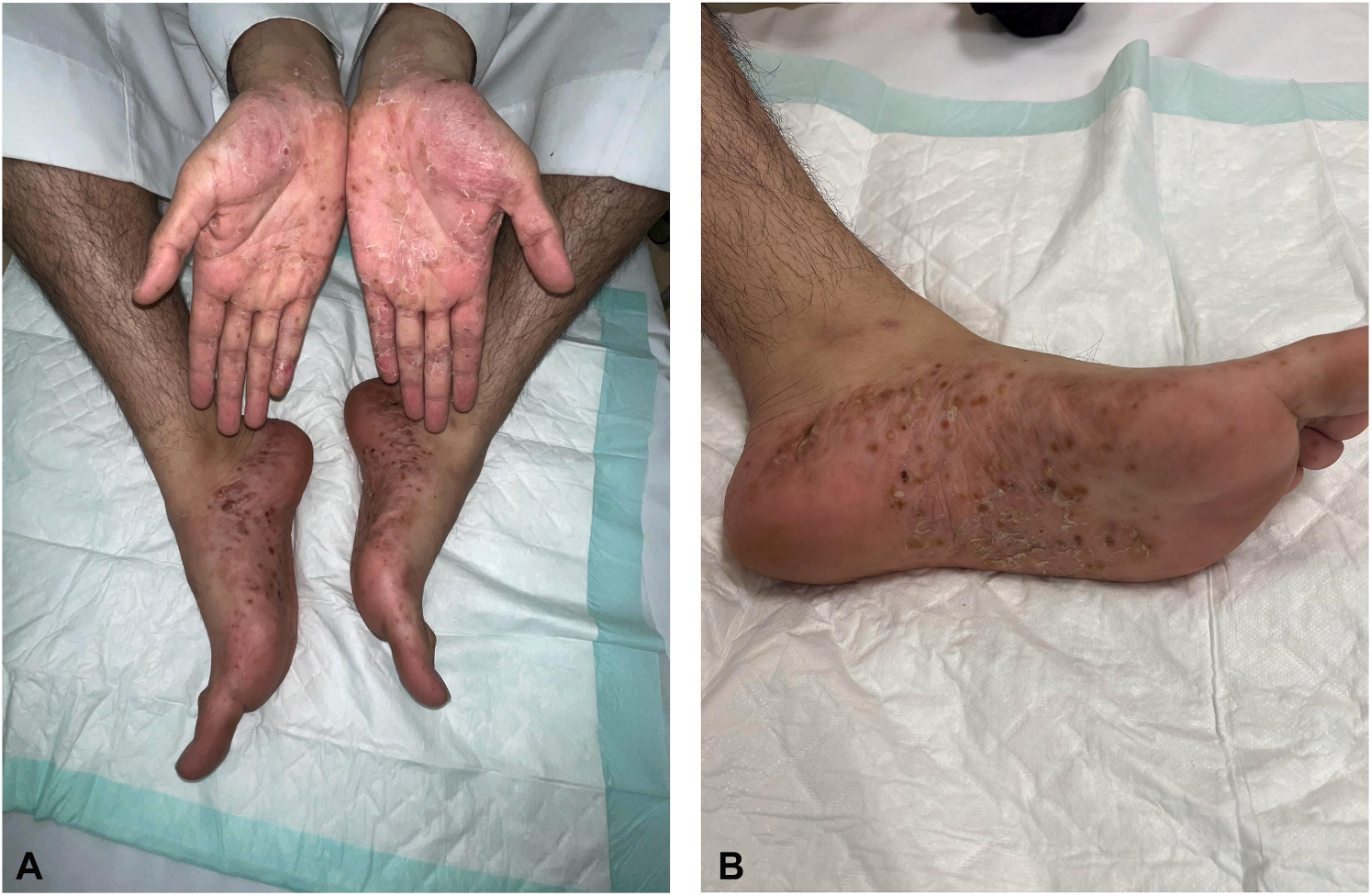
**A,** Symmetrical erythematous, scaly plaques with superficial pustules involving the palms and soles. **B,** Close-up of the right plantar surface showing grouped pustules and crusting on a hyperkeratotic background.

Due to the severity and chronicity of his disease, secukinumab was initiated according to the standard loading regimen (300 mg subcutaneously at weeks 0, 1, 2, 3, and 4, followed by monthly dosing). Four weeks after starting the medication (after the fifth dose), the patient developed new erythematous, scaly plaques with superficial sterile pustules confined to the palms and soles (Fig 1, *A* and *B*). These lesions were painful and pruritic but were not accompanied by fever or systemic symptoms. No new medications or topical agents had been introduced.

**Fig 2:**
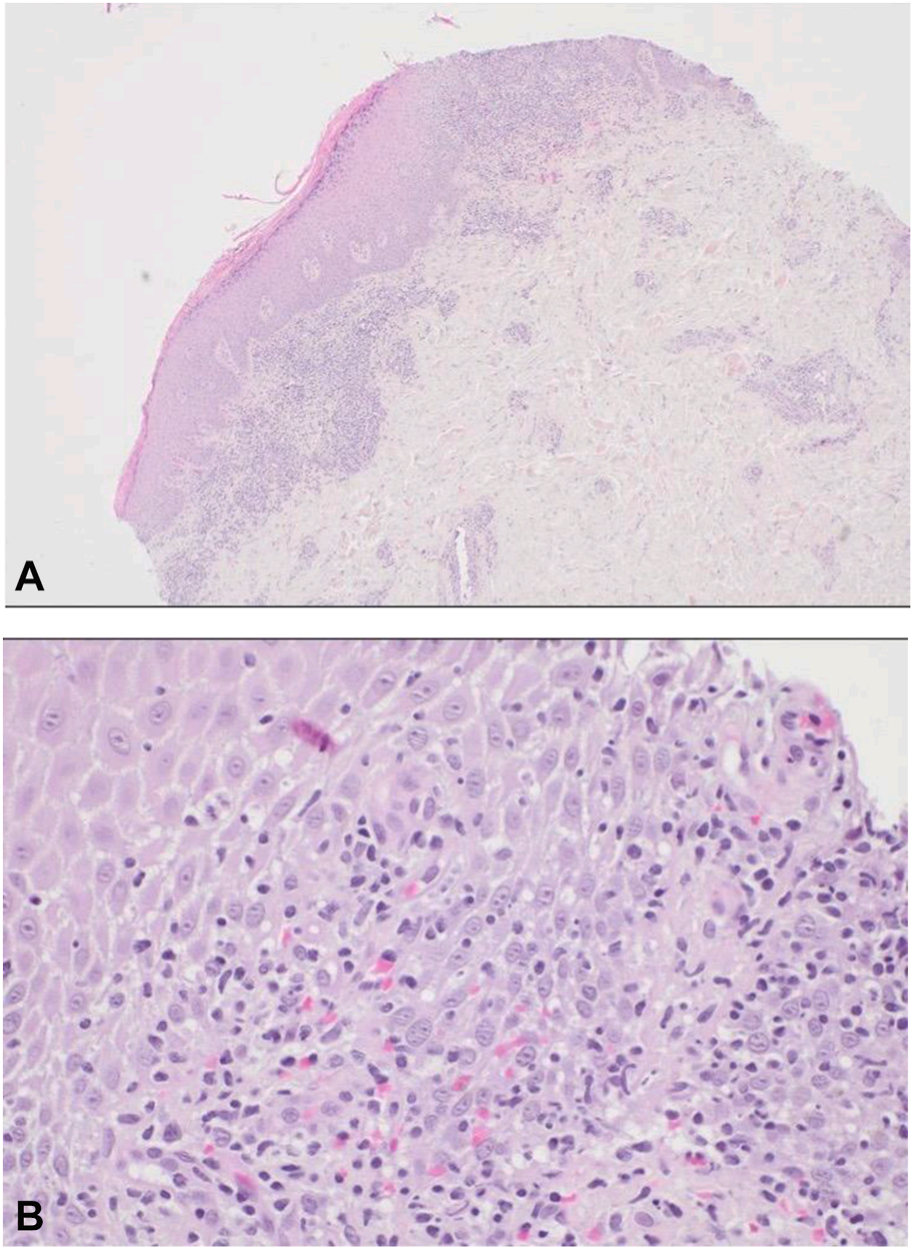
Histopathologic features from a punch biopsy of the plantar lesion showing spongiosis with acanthosis and superficial perivascular lymphocytic infiltrate with scattered eosinophils. Gomori methenamine silver stain was negative for fungal elements. **A,** Low-power view showing spongiosis and acanthosis. **B,** High-power view showing superficial perivascular lymphocytic infiltrate with scattered eosinophils.

On examination, symmetrical erythematous plaques with overlying scale and pustules were observed on both palms and soles (Figs 1, *A*, *B* and 2 *A*, *B*) highly suggestive of PPP. A punch biopsy was performed; however, histopathologic findings were nonspecific and did not demonstrate classic psoriasiform changes. Given the acral distribution, pustular morphology, temporal association with secukinumab, and absence of alternative triggers, a paradoxical PPP secondary to IL-17 inhibition was diagnosed.

Secukinumab was discontinued. The patient was started on topical clobetasol propionate with gradual clinical improvement. Due to persistent HS activity, adalimumab was initiated (160 mg loading dose followed by 40 mg weekly), resulting in improvement of both HS and acral lesions. At the 3-month follow-up visit, pustules had resolved, with only mild residual erythema and scaling. Apremilast was considered as a potential future systemic treatment option.

## Discussion

Paradoxical cutaneous reactions have been increasingly reported in patients receiving biologic therapies, particularly IL-17 inhibitors such as secukinumab. Although these agents are widely used for psoriasis and psoriatic arthritis and are being investigated for HS, they have been implicated in inducing new-onset or worsening inflammatory dermatoses. The most frequently reported are pustular psoriasis and PPP, although eczematous eruptions have also been described.^1^, ^2^, ^3^

In this case, the patient developed classic acral pustular lesions consistent with PPP after the fifth dose of secukinumab. Despite nonspecific histopathologic findings, the characteristic acral distribution, pustular morphology, and temporal relationship with IL-17 inhibition strongly supported the diagnosis. This underscores the importance of clinicopathologic correlation, as paradoxical PPP may demonstrate spongiosis and mixed inflammatory infiltrates rather than classic psoriasiform changes.^1^^,^^2^

The exact pathogenesis is not fully understood; however, IL-17 inhibition may disrupt immune homeostasis by suppressing Th17 signaling while promoting compensatory upregulation of alternative pathways such as interferon-α, IL-36, and IL-22.^4^ This immune shift can trigger PPP in genetically or immunologically predisposed individuals. A systematic review by Mössner and Pinter reported that approximately one-third of paradoxical reactions to IL-17 blockade presented as PPP.^1^ Massone et al similarly reported PPP cases associated with secukinumab and brodalumab, which resolved upon drug withdrawal.^2^

Patients with HS may be particularly susceptible to paradoxical immune phenomena due to the underlying dysregulation of cytokines such as TNF-α, IL-1β, IL-17, and IL-23.^5^ Inhibiting IL-17A may further shift this cytokine network and precipitate PPP.

In our patient, discontinuation of secukinumab and initiation of adalimumab led to improvement of both HS and PPP, consistent with prior reports that switching to another biologic class—particularly TNF-α or IL-23 inhibitors—can successfully manage both the primary disease and the paradoxical reaction.^1^ Adjunctive topical corticosteroids, calcineurin inhibitors, and systemic doxycycline supported clinical resolution. Similar cases have been reported in the literature.^6^

## Conclusion

This case highlights PPP as a rare but clinically relevant paradoxical reaction to IL-17 inhibitor therapy in patients with HS. The appearance of new acral pustules in patients receiving secukinumab should raise suspicion for PPP. Early recognition, careful clinicopathologic correlation, and timely discontinuation of the offending agent are vital. Switching to an alternative biologic class, such as TNF-α inhibitors, may allow control of both the underlying disease and the paradoxical eruption.

### Declaration of generative AI and AI-assisted technologies in the writing process

AI-assisted technology was used only for limited language refinement, phrasing, and readability. It was not used to generate or alter any clinical data, patient information, images, scientific findings, interpretation, or conclusions. All scientific content was reviewed and approved by the authors, who take full responsibility for the manuscript.

## Conflicts of interest

None disclosed.
